# Literary and Miscellaneous

**Published:** 1890-08

**Authors:** 


### Literary and Miscellaneous.

PERHAPS the most extraordinary article ever published upon Hypnotism, appears in the *Cosmopolitan Magazine* for August. It was secured from one of the two most celebrated professors of the weird art, the Frenchman Donato, and the illustrations were secured by having a number of subjects taken to the photograph gallery of Mr. Kurtz, in New York, and there hypnotised under the camera by Donato himself. The illustrations show very fairly the frightful powers which the hypnotiser exerts; and the whole article helps to make plain a subject which is now exciting much attention all over the world. One who has not seen the facile movements of the hypnotiser and the change which takes place in the victim under his apparently simple action, cannot for a moment comprehend the wonderful powers exercised. One moment the subject looks you in the eyes, talks to you as any other person; is in his right mind in every particular; the next, under a motion of the professor, his mind is as completely lost to his body as if his head had been cut off, and in this condition, subject to suggestions of the operator,—suggestions which may be carried to the most farcical or the most terrible results,—he remains until recalled to life by the hypnotiser. Never before has a number of subjects been placed under the camera and operated upon in this way, and the article will doubtless be received with general interest throughout the country.

---

A NEW index of current medical literature has appeared under the following title:

Revue Internationale de Bibliographie Médicale, Pharmaceutique et Vétérinaire. Dirigée par le Docteur Jules Rouvier, Professeur de clinique obstetricale et gynecologique á la Faculté francaise de médicine de Beyrouth, etc., etc. Paris, Librairie Medicale, Vue Jacques Lechevalier 23, Rue Racine.

The first number of this work appeared in April, and the second is now before us; like the first number, it goes over the field of medical literature with much tact and accuracy.. Its exchange list comprises nearly 200 of the ablest medical journals of the world—thirty-two belonging to the United States. It is published quarterly, and sells for the modest sum of ten francs ($2.00). Every physician who is unable to keep the Index Medicus on his table, will find in this work a worthy substitute at one-fifth the cost of the American publication. The print is plain, titles of papers and authors being in large-faced type, making it as convenient to refer to as such a work possibly can be.

---

THE following circular letter has been issued to the profession:

The Erie County Eye, Ear, and Throat Infirmary, corner of Main and East Huron streets, is now open for the gratuitous treatment of indigent patients.

In starting this new institution for the treatment of diseases of the eye, ear, nose, and throat, we respectfully ask your coöperation in our endeavors to carry it on in a manner which shall meet the approbation of the members of our profession.

When persons who say that you are their family physician apply to us, we intend to refer them to you for a note which shall state that you approve of their seeking our aid. We shall always be pleased to treat such patients as you may send to us as needing special skill or apparatus for their relief, and, whenever you desire it, will send you a written statement of the conditions which we find. As this is a purely charitable institution, however, we trust that you will not recommend it to such patients as are able to pay for medical services.

Members of the profession and medical students are always welcome at the Infirmary, and we hope to make the large amount of clinical material which is already collecting here, of much use for the advancement of medical science.

Very truly yours,

FRANK W. ABBOTT, M. D.,<br>

BENJAMIN H. GROVE, M. D.,<br>

F. WHITEHILL HINKEL, M. D.,<br>

} *Surgeons in Charge.*

---

AN INVITATION to contribute to the erection of a monument to the memory of the late Richard von Volkmann, Professor of Surgery in Halle, has been issued by a committee composed of eminent men of the medical and other professions in the old and the new world. It reminds the reader that the great eminence in science and fiction of von Volkmann has inspired his friends to erect a monument to his memory. It speaks in a forcible and touching way of his marvelous ability as a surgeon, his eminent gift of teaching and writing, which educated so many skilful disciples, of his poetry and fictitious prose, of his faith in his king and country, his humility toward God, and his love of high ideals. The coöperation of all people is asked for, high and low, rich and poor, at home and abroad. The monument will be erected in or before the clinic of which von Volkmann was the director. Contributions can be sent to any member of the business committee, or to the treasurer, Mr. Dehne, Commerzienrath in Halle (Saale), Germany. It is to be hoped that this appeal will meet with a generous response on the part of all who have known or felt the influence of this undisputed master of surgery.

---

THE Alvarenga Prize, of the College of Physicians of Philadelphia, consisting of one year's income of the bequest of the late Senor Alvarenga, of Lisbon, has been awarded to Dr. R. W. Philip, of the Victoria Dispensary for Consumption and Diseases of the Chest, Edinburgh, for his Essay on Pulmonary Tuberculosis, which will be published by the college.

---

THE Therapeutical Apparatus of Peroxide of Hydrogen (medicinal) and Glycozone, is the title of a neat and useful pamphlet published by Charles Marchand, chemist, a graduate of the Central School of Arts and Manufactures of Paris. In this brochure is considered the treatment of diseases caused by germs, bacteria, and microbes.

